# Large language models improve clinical decision making of medical students through patient simulation and structured feedback: a randomized controlled trial

**DOI:** 10.1186/s12909-024-06399-7

**Published:** 2024-11-28

**Authors:** Emilia Brügge, Sarah Ricchizzi, Malin Arenbeck, Marius Niklas Keller, Lina Schur, Walter Stummer, Markus Holling, Max Hao Lu, Dogus Darici

**Affiliations:** 1 Connectome – Student Association for Neurosurgery, Neurology and Neurosciences, Berlin, Germany; 2 Department of Neurosurgery, University Hospital of Münster, Münster, Germany; 3 Harvard Graduate School of Education, Cambridge, USA; 4 Institute of Anatomy and Neurobiology, University of Münster, Vesaliusweg 2-4, 48149 Münster, Germany

**Keywords:** Large language models, Clinical decision making, Medical students education, Structured feedback, Patient simulation training

## Abstract

**Background:**

Clinical decision-making (CDM) refers to physicians’ ability to gather, evaluate, and interpret relevant diagnostic information. An integral component of CDM is the medical history conversation, traditionally practiced on real or simulated patients. In this study, we explored the potential of using Large Language Models (LLM) to simulate patient-doctor interactions and provide structured feedback.

**Methods:**

We developed AI prompts to simulate patients with different symptoms, engaging in realistic medical history conversations. In our double-blind randomized design, the control group participated in simulated medical history conversations with AI patients (control group), while the intervention group, in addition to simulated conversations, also received AI-generated feedback on their performances (feedback group). We examined the influence of feedback based on their CDM performance, which was evaluated by two raters (ICC = 0.924) using the Clinical Reasoning Indicator – History Taking Inventory (CRI-HTI). The data was analyzed using an ANOVA for repeated measures.

**Results:**

Our final sample included 21 medical students (age_*mean*_ = 22.10 years, semester_mean_ = 4, 14 females). At baseline, the feedback group (mean = 3.28 ± 0.09 [standard deviation]) and the control group (3.21 ± 0.08) achieved similar CRI-HTI scores, indicating successful randomization. After only four training sessions, the feedback group (3.60 ± 0.13) outperformed the control group (3.02 ± 0.12), F (1,18) = 4.44, *p* = .049 with a strong effect size, partial *η*^2^ = 0.198. Specifically, the feedback group showed improvements in the subdomains of CDM of creating context (*p* = .046) and securing information (*p* = .018), while their ability to focus questions did not improve significantly (*p* = .265).

**Conclusion:**

The results suggest that AI-simulated medical history conversations can support CDM training, especially when combined with structured feedback. Such training format may serve as a cost-effective supplement to existing training methods, better preparing students for real medical history conversations.

**Supplementary Information:**

The online version contains supplementary material available at 10.1186/s12909-024-06399-7.

## Introduction

Clinical decision-making (CDM) refers to the ability of healthcare professionals to gather, evaluate, and interpret relevant information [1]. An integral component of CDM is the medical history conversation, traditionally practiced on real or standardized patients combined with feedback on the performance [2]. Thereby, CDM is highly intertwined with the concept of clinical reasoning, which refers to the cognitive process that healthcare professionals use to understand and process patient information, recognize clinical patterns, and evaluate evidence to reach a diagnosis and determine an appropriate treatment plan [3].

Conversations with real patients, or simulated patients are the traditional approaches for training clinical-decision-making. These training interactions are designed to teach and assess skills such as history taking, physical examination, and communication, often involving trained actors who simulate patient scenarios. However, training with real patients or human actors is often costly and limited, reducing the availability of these simulation opportunities for students [4, 5].

Various technologies have been adopted to expand the availability of simulations, with the goal to make it more accessible to more students. For example, Virtual Reality (VR) and Augmented Reality (AR), along with various forms of computer-based simulations, have been used to simulate different CDM situations [6].

### LLM-simulated CDM-training

One emerging technology with the potential to make CDM even more accessible is the use of LLMs, such as ChatGPT. Kung et al. showed that ChatGPT (2022 December version) can successfully pass the United States Medical Licensing Exam (USMLE), showing its “knowledge” in medical area [7]. In addition, it has been shown that LLM can successfully mimic patients with various types of medical history or personality, and can be a valuable tool for educating patients in a clinical setting [8–11]. Therefore, and with the ability to specify context for each conversation, LLMs may be able to simulate natural discussions with students, and be used as a cost-effective way to train CDM skills.

However, the use of LLMs have also been associated with poorer outcomes in some cases [12]. Inaccurate information produced by LLMs can lead to misconceptions and faulty knowledge construction, and when the answer lacks proper instruction, it could mislead the user rather than providing clarifications. Therefore, there is currently a need for empirical studies that carefully design and evaluate the use of LLMs in CDM-training.

### Incorporating LLM-generated feedback

A critical pedagogical consideration in designing LLMs for CDM training is the role of feedback. Well-founded feedback after completing a task is essential for an effective learning process [13]. For many years, research into educational sciences has focused on the topic of feedback culture and its impact on learning outcomes [13, 14]. It is widely acknowledged that getting feedback after a performance helps identify areas of strength and improvement while reinforcing good practices [15, 16]. Studies have shown that feedback in online learning environments can improve student performance in about 65% of cases [17]. The most significant impact occurs when students receive immediate, and task-specific feedback along with strategies for more effective task completion, rather than general praise or criticism [18–20].

Among the few studies that exist on LLM-generated patient simulations [21–24], none have implemented feedback or debriefing at the time of reporting. Therefore, we were particularly interested to investigate to what extent automatically generated feedback from LLMs after a patient simulation can improve students’ performance.

## The current study

To address these gaps in the literature, we designed a set of LLM prompts that simulate patients with different symptoms and medical histories. Afterward, medical students were randomly assigned into two groups to conduct CDM activities with the simulated patient, while only one group received feedback after their simulated conversation.

We hypothesized that the group that receives additional feedback would achieve better CDM scores than the control group that received no additional feedback. Furthermore, we hypothesized that the participants perceive the LLM-based CDM training as helpful and realistic.

## Methods

We conducted a two-group randomized-controlled study during winter term 2023/2024 at the University of Münster, Germany and used the large language model OpenAI’s ChatGPT 3.5 (“generative pretrained transformer”), free version. Ethics approval was obtained from the ethics board (“Ethik-Kommission Westfalen-Lippe”) under the reference 2023-438-f-N. Informed consent was obtained from all participants. A clinical trial number was not applicable.

### Study population

For the study, we recruited medical students from the University of Münster using an e-Mail distribution list between November 2023 and February 2024. The study was promoted in lectures. Participants had to be enrolled as medical students to be included. In the following sections, the medical students who were included in the study population will be referred as participants. Data was collected anonymously, and all participants took part in the study voluntarily. No allowance was paid.

The following inclusion criteria were applied: (1) Participants needed to be enrolled medical students of the University of Münster. (2) Participants needed to attend the study on site in person. (3) Participants needed to give full consent.

### Study procedure

**Fig. 1:**
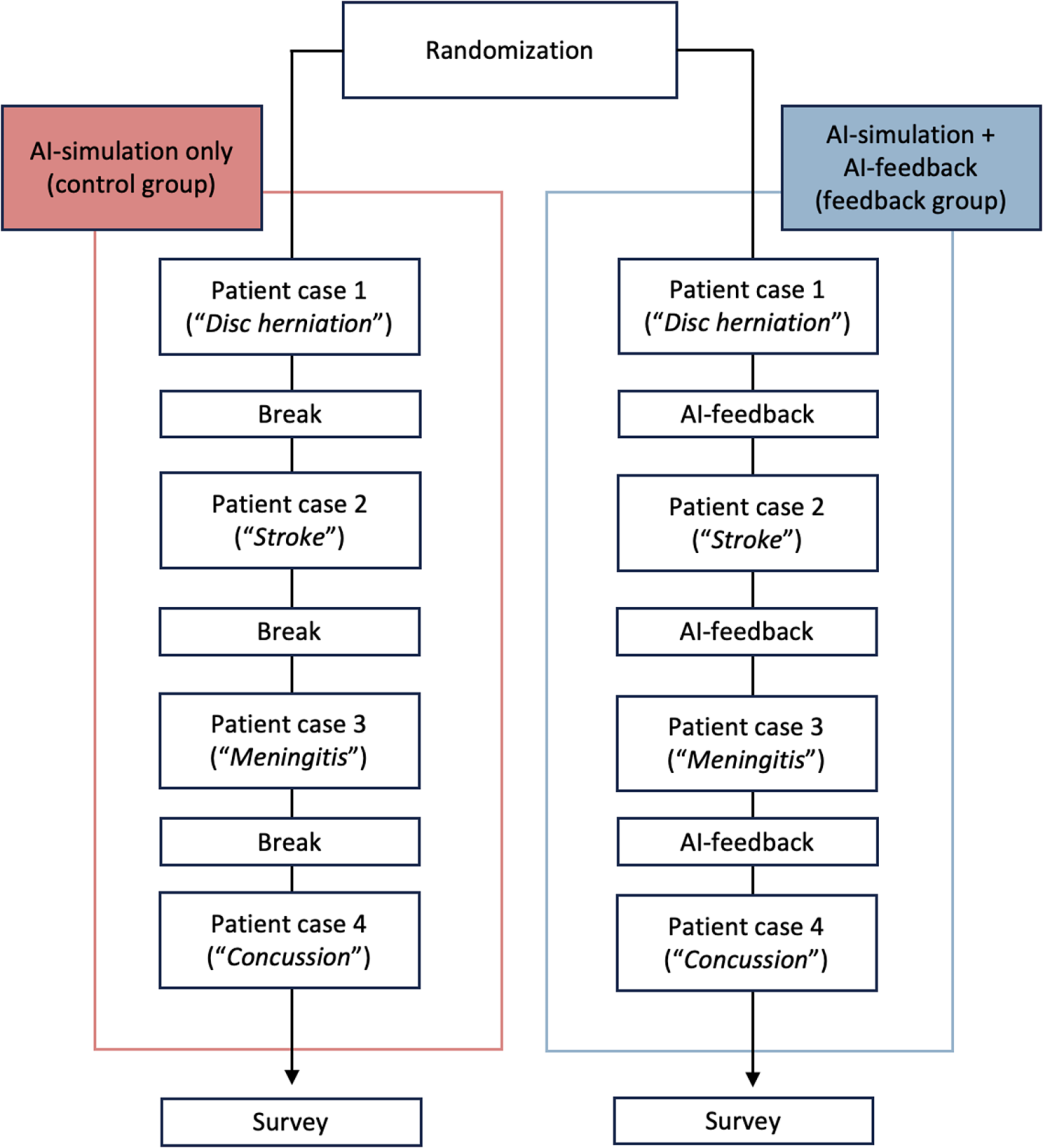
Study design. The diagram illustrates the timeline of the study. The control group completed four patient cases, with a 2-minute break between each case. In contrast, the feedback group received feedback from ChatGPT during these breaks instead of pausing. After fulfilling the tasks, the participants received a survey with no time limit

For the study execution (Fig. 1), participants were randomly assigned (per *simple random allocation*) to one of two groups: a control group (AI-simulated patient conversation only) and a feedback group (AI-simulation patient conversation and feedback). Participants were blinded to the intervention (closed-label RCT). Both groups received the same prompt. In addition to the conversation, the feedback group received feedback from ChatGPT after each simulated conversation (see below). Both groups had the same number of training opportunities, with no difference in the order and frequency of patient case scenarios. The AI-generated feedback focused solely on CDM processes rather than on transmitting medical content.

The study was conducted on the premises of the medical faculty of the University of Münster. After randomization, the research team placed the participants at prepared workplaces.

The simulated conversations were conducted within the ChatGPT Playground Interface. We created contextual prompts (see below for a detailed explanation) simulating a neurological or neurosurgical emergency situation in a chat window. These simulated patient cases reflected the CDM tasks. The tasks involved conducting a thorough medical history conversation to develop a differential diagnosis for the patient’s condition, a common CDM practice. The AI takes on the role of a patient presenting symptoms and engaging in a realistic medical history conversation with a study participant.

The corresponding contextual prompt (see below) was entered into AI before the study participants arrived. To ensure that participants could only view the conversation interface, parts of the computer screen were covered. There was no opportunity for the study participants to communicate with each other while performing the tasks, as adequate distance was ensured between the workstations. Three researchers monitored the participants during the execution of the tasks. Predetermined times were announced out loud for all participants.

The control group conducted the conversation with the simulated patient independently, while the feedback group received mentoring feedback from ChatGPT after completing each task. For each patient case, the participants had six minutes to take a medical history of the simulated patient with pre-specified conditions in order to suspect a diagnosis.

### CDM task

Participants were asked to practice CDM in four scenarios in which ChatGPT simulates patients with the following diagnosis: (1) herniated lumbar disc, (2) stroke, (3) meningitis and (4) concussion. The selection of scenarios was made in consultation with the department of neurosurgery of the university hospital of Münster, ensuring both medical accuracy of the clinical context and practicality of the training tasks.

### Prompt for control group

The prompt consisted of four different patient cases (Table 1). Both groups were given the same CDM tasks to complete which was to suspect a diagnose for the patient by having a medical history conversation. Across both groups, ChatGPT received basic contextual prompts regarding the patient cases (see Table 1).

**Table 1:** AI-prompts used to simulate clinical decision-making scenarios as exemplified by one scenario

Prompt for control group (AI simulation only)	Prompt for feedback group (AI simulation + *AI feedback*)
Scenario (4): Traumatic Brain Injury I would like you to behave as if you were simulating a patient who comes into the emergency room with a traumatic brain injury. • You should simulate the patient as if you were talking to a doctor. • Answer sparingly and only to the questions asked. • You do not know that you have a traumatic brain injury. • Respond from the patient’s perspective. • Have the patient answer very vaguely and imprecisely. The user will simulate the doctor and respond to your comments and ask further questions. You always answer only after the doctor has asked you a question. The user will end the conversation with the command “END”. Before we begin, please confirm that you have understood the request with the word “BEGIN”. Afterward, please simulate the first message of the patient. You introduce yourself and explain why you have come to the emergency room. You are the patient. The user is the doctor.	Scenario (4): Traumatic Brain Injury I would like you to behave as if you were simulating a patient who comes into the emergency room with a traumatic brain injury. • You should simulate the patient as if you were talking to a doctor. • Answer sparingly and only to the questions asked. • You do not know that you have a traumatic brain injury. • Respond from the patient’s perspective. • Have the patient answer very vaguely and imprecisely. The user will simulate the doctor and respond to your comments and ask further questions. You always answer only after the doctor has asked you a question. The user will end the conversation with the command “END”. *At this point*,* you will provide feedback on how the user*,* in their role as a doctor*,* could improve the anamnesis.* • *Your feedback should include the following eight criteria*: 1. *Assess whether the user has taken control of the conversation to obtain the necessary information.* 2. *Assess whether the user recognizes all relevant information.* 3. *Assess whether the user formulates targeted questions so that he can capture and specify the symptoms in detail.* 4. *Assess whether the questions of the user suggest that specific causes or circumstances lead to certain symptoms.* 5. *Assess whether the user asks questions in a logical sequence.* 6. *Assess whether the user reassures the patient that he has received the correct information from the patient.* 7. *Assess whether the user has summarized his collected information before ending the conversation.* 8. *Assess whether the user has collected sufficient information of high quality at an appropriate speed.* *Assign each of the eight criteria a score according to the following scheme*: *1 - Does not meet the criterion 2 - Rather does not meet the criterion 3 - Partially meets the criterion 4 - Rather meets the criterion 5 - Fully meets the criterion Explain the evaluation with two sentences.* *Create three suggestions for improvement in bullet points aimed at strengthening clinical reasoning skills.* *End the feedback with the word: “END”.* Before we begin, please confirm that you have understood the request with the word “BEGIN”. Afterward, please simulate the first message of the patient. You introduce yourself and explain why you have come to the emergency room. You are the patient. The user is the doctor.

Note. The feedback ground (right column) received additional feedback from ChatGPT. The prompt that generates the feedback is highlighted in italic

The prompt was designed by refining different contextual prompts and evaluating the corresponding interactions with ChatGPT. Through multiple pilot phases, we optimized the prompts to provide responses that simulate a realistic knowledge level and react appropriately to unusual queries or requests. Through the final selected prompts, we were able to obtain meaningful answers to relevant medical history questions from ChatGPT.

### Prompt for feedback group

Recognizing the importance of feedback in medical history training, we enhanced the prompt for the feedback group with AI-generated feedback for clinical decision-making (see Table 1, blue text). The CDM was qualitatively assessed by ChatGPT using the Clinical Reasoning Indicator-History Taking Inventory score (CRI-HTI) [25]. This is done by adding additional information into the contextual prompt we provided to ChatGPT at the beginning. In addition to the assessment, the prompt also asked ChatGPT to generate three personalized suggestions for improvements in bullet points aimed at strengthening clinical reasoning skills after every patient case.

### Measures

The CRI-HTI, developed and validated by Fürstenberg et al. (2016) [25], measures the clinical reasoning skills of medical students using a five-point Likert-Scale. The score consists of eight criteria assigning to three fields of competence: “Focusing questions”, “Creating context” and “Securing information”. The following items were included: “1) Taking the lead in the conversation 2) Recognizing and responding to relevant information 3) Specifying symptoms 4) Asking specific questions that point to pathophysiological thinking 5) Putting questions in a logical order 6) Checking with the patient 7) Summarizing 8) Collected data and effectiveness of the conversation”.

The criteria were translated to German and entered as part of the contextual prompt to ChatGPT. In addition, ChatGPT was asked to rate the conversation according to the following pattern: “Assess whether the user has taken control of the conversation to obtain the necessary information”. Each item is evaluated according to a five point Likert-Scale that ranges from 1 (does not meet the criterion) to 5 (fully meets the criterion). An increase of 1 point in the scale equals an improvement of 20%. The CRI-HTI was validated in German language with German medical students with a high internal consistency of 0.78. The usage of a scale validated in German language minimizes the potential bias from reduced validity due to translation. After the training, the perceived realism, authenticity and accuracy of the simulation was assessed using a survey.

### Data analysis

**Fig. 2:**
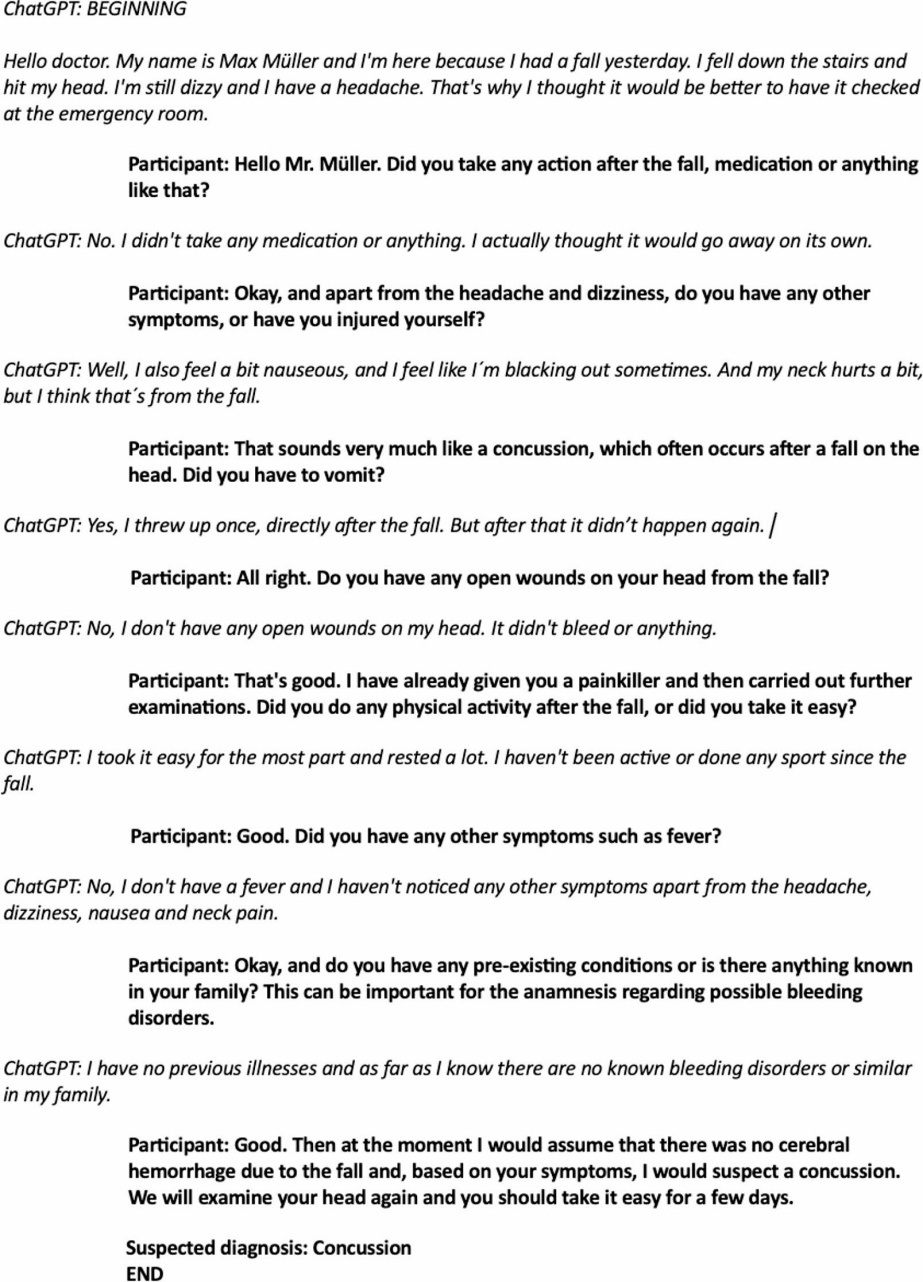
Example of a conversation between the participant and ChatGPT. The prompt of the fourth scenario instructed ChatGPT to simulate a patient with a concussion. Italicized text represents the responses given by ChatGPT

**Fig. 3:**
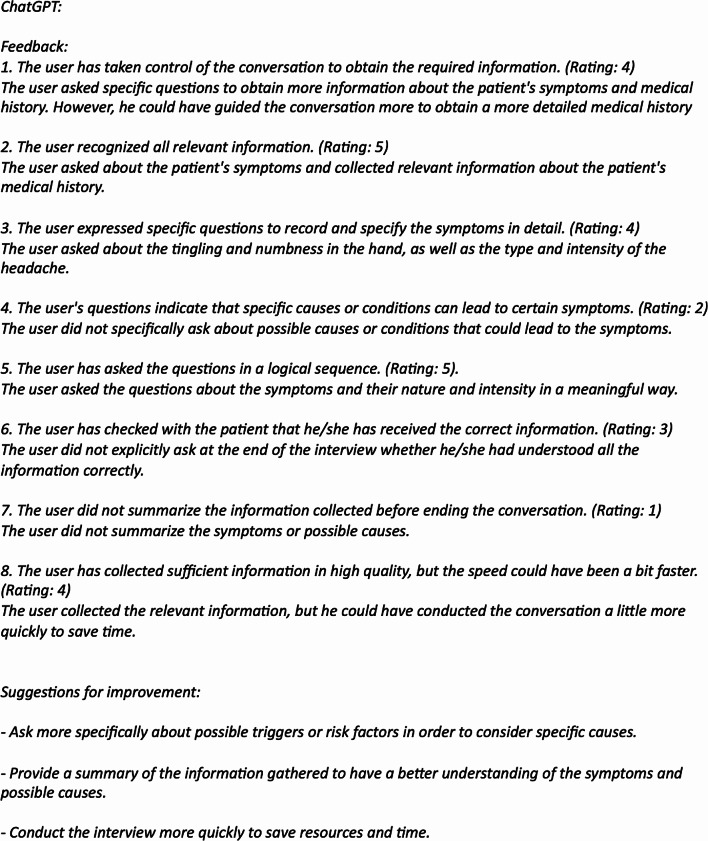
Example of the feedback using the CRI-HTI-Score and the individual feedback given by ChatGPT. The participant received the feedback after completing scenario 2. This is the same participant as in Fig. 3. Italicized text represents the responses given by ChatGPT

After the completed data collection, two independent raters read the participants’ chat conversations and assessed their clinical reasoning ability according to the CRI-HTI. The raters were blinded, i.e. had no knowledge of which group each participant belongs to, and showed an excellent agreement, ICC = 0.924. An example of the simulated conversation is shown in Fig. 2. Standardized feedback is shown in Fig. 3.

### Statistical method

The data was analyzed using SPSS (Version 28, IBM Corp., Armonk, NY). To compare the progression of the CRI-HTI over time (3 levels), we performed an ANOVA for repeated measures with the CRI-HTI as a within-group and the group allocation (feedback group vs. control group) as between-group factors. Gender, age, and semester were included as covariates. All statistics were performed two-sided under a significance level of *α* = 0.05. The statistical testing results were specified by an effect size with partial *η*^2^ = 0.01 considered a small, *η*^2^ = 0.06 a moderate, and *η*^2^ = 0.14 a strong effect.

The intraclass correlation coefficient (ICC) was used to assess the reliability of CRI-HTI ratings across the two human raters. A two-way mixed-effects model with 95% confidence intervals was applied focusing on the consistency of ratings rather than absolute agreement, as we were primarily interested in whether raters rank individuals similarly rather than achieving exact numeric matches.

## Results

### Study population

In total, *N* = 29 students initially agreed to participate in the study. Before randomization, 5 students dropped out by not attending the study session, leaving 24 students who began the study and underwent randomization. The final analysis included 21 participants, as we had to exclude 3 participants due to technical difficulties where their data was not properly saved during the upload process. These drop-outs were unrelated to either the intervention or any participant characteristics (“missing completely at random”). The final study group included 21 participants between the ages of 19 and 35. The control group consisted of 11 participants and 10 participants for the feedback group. None of the participants had clinical rotations so far, and half of the population answered to have no prior knowledge. The majority was in 3rd semester and 21 years old.

When comparing their sociodemographic information, no group differences were observed, indicating tentative successful randomization (Table 2). Specifically, neither age, gender, prior knowledge nor semester of study showed a significant difference between the two study groups. No participants had extended medical knowledge on the topic of neurology or neuroscience such as internships, lectures or modules.

**Table 2:** Participant characteristics after simple random allocation

Variable	Feedback group *n* = 10	Control group *n* = 11	Significantly different?
Age (years)			*χ*2(6) = 7.58, *p* = .271
19	0	1	
20	1	2	
21	8	3	
22	0	2	
23	0	1	
26	1	1	
35	0	1	
Gender			*χ*2(1) = 0.095, *p* = .757
* Male*	3	4	
* Female*	7	7	
* Diverse*	0	0	
Prior knowledge			*χ*2(3) = 0.649, *p* = .885
* Without prior knowledge*	5	5	
* Non-clinical internship*	3	3	
* Clinical internship*	1	0	
* Clinical rotation*	0	0	
* Other*	2	4	
Semester of study			*χ*2(3) = 3.560, *p* = .313
* 3*	6	9	
* 6*	3	1	
* 7*	0	1	
* 8*	0	1	

Note. No significant group differences were observed, indicating successful randomization

To further ensure group equivalence at baseline, we compared the pre-intervention word, using the chat transcript the students had with the AI, during scenario 1 between the feedback group (*Mean* = 86.89, *SD* = 20.02) and control group (*Mean* = 85.09, *SD* = 16.67). A *t*-test for independent samples showed no significant difference between the groups, t(18) = 0.219, *p* = .829, indicating that both groups were comparable at baseline.

### Effects of feedback on performance

**Fig. 4:**
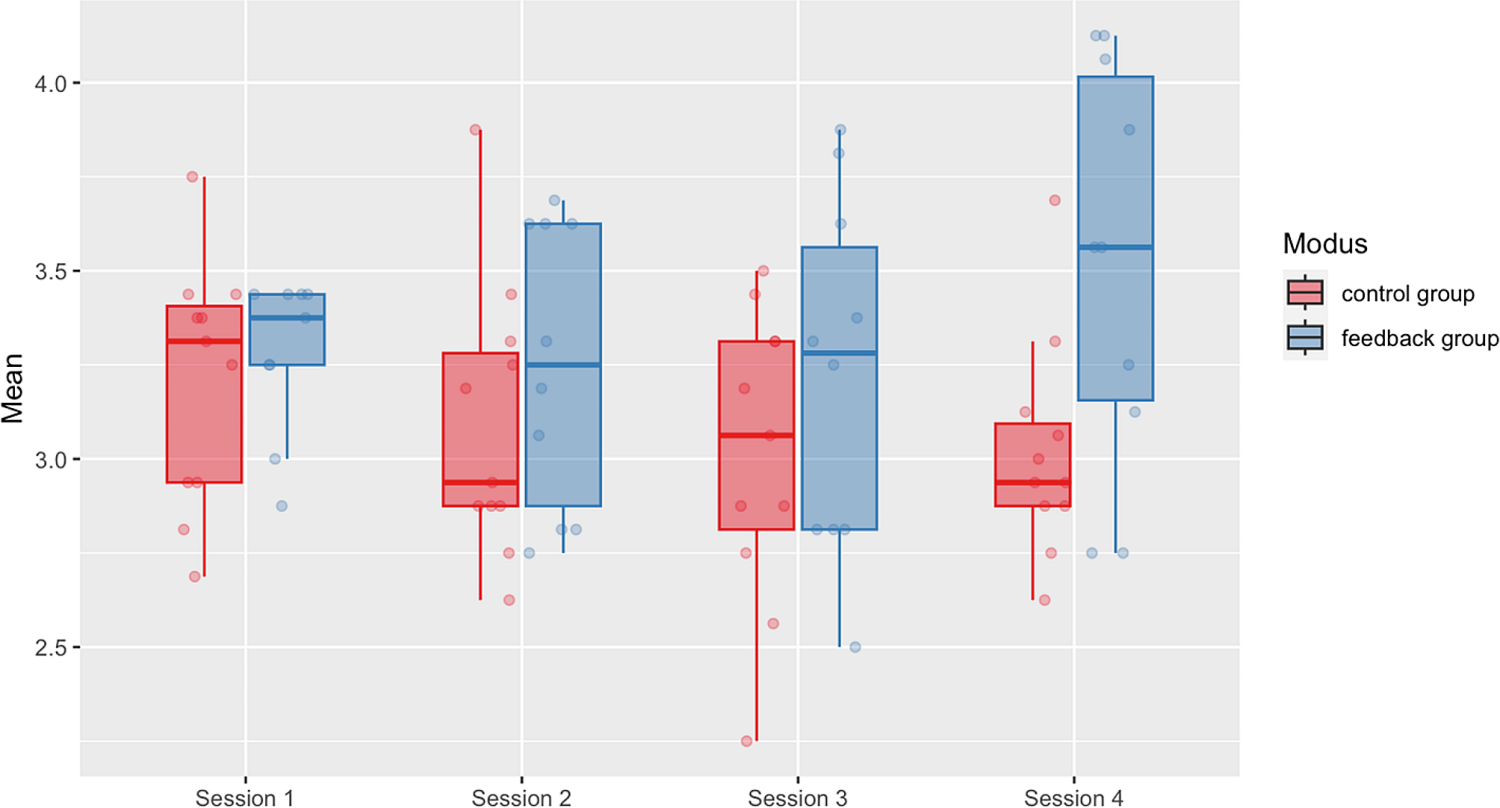
Effects of AI-generated feedback on clinical decision-making and history taking. Median CRI-HT Score (ICC = 0.924) with the individual values is shown for the control group (red) and feedback group (blue) over four subsequent AI-simulated history taking scenarios

A repeated measures ANOVA was performed to evaluate the effects of the feedback provided by ChatGPT on the performance of the participant. The results demonstrated significantly better outcomes in the feedback group, with participants showing a steady improvement from scenario 1 to scenario 4 compared to the control group (Fig. 4). After four scenarios, participants in the feedback group (*M* = 3.604 ± 0.130; 95% *CI*: 3.331, 3.878) outperformed the control group (*M* = 3.017 ± 0.118; 95% *CI*: 2.770, 3.264), in CDM, F(1,20) = 4.436, *p* = .049, with a strong effect size, partial *η*^2^ = 0.198.

Specifically, the feedback group showed improvements in the CDM-subdomains creating context (*p* = .046) and securing information (*p* = .018), while their ability to focus questions did not improve significantly (*p* = .265). Together, the results indicate that AI-based medical history conversations with feedback can simulate conducive teaching and learning scenarios, and improve students’ CDM performances. We also screened the feedback provided for its quality, with most instances being deemed specific, realistic, and helpful (Supplemental Fig. 1). Furthermore, the participants in both groups rated the simulations as being highly accurate (*M* = 8.67/10 ± 2.01), feasible (*M* = 8.38/10 ± 1.88), and realistic (*M* = 7.81/10 ± 1.40).

## Discussion

This study is the first empirical study to show that automatically generated feedback by Large Language Models (LLM) can enhance the clinical decision-making (CDM) training effects for medical students in LLM-simulated conversations. Using an experimental design, we found that the group receiving LLM-generated feedback demonstrated a significant improvement across four clinical scenarios compared to the control group. The strong effect size indicates that this effect is practically relevant. This finding holds potential to transform the way we teach CDM, particularly for conducting medical history conversations.

### The impact of LLM-generated feedback on CDM training

Our results align with the vast amount of research that underscores the importance of feedback in medical training. The students in the feedback group showed significant improvements in the subdomains of CDM, such as “creating context”, and “securing information”. LLM-generated feedback may have helped the students to reflect on their skills and adapt more quickly. In a similar vein, other comparative studies have shown that feedback from LLMs is perceived as equally beneficial as human-generated feedback [26]. This indicates that LLMs can generate immediate, specific feedback that helps students refine their questioning techniques and better gather relevant patient information (Figs. 2 and 3).

The integration of LLM-based feedback offers students an additional, readily available tool for medical training and skill development. Our findings demonstrate that the feedback provided by ChatGPT was perceived as helpful by participants and had a positive effect on their performance. In the future, it would be interesting to examine the effect over a longer period, with regular use of ChatGPT integrated into the daily studies of medical students.

### Effects on the subdomains of CDM

We further observed that the feedback group demonstrated varying levels of improvement across the three subdomains of CDM, with the most notable progress in “securing information”. We think that the pronounced improvement in this particular subdomain likely stems from several factors inherent to both the nature of information gathering and the feedback process itself. Information securing represents a concrete, observable skill that can be readily identified and refined through specific feedback. When conducting patient interviews, students must learn to systematically collect relevant information, and feedback can directly highlight missed opportunities, suggest alternative questions, or reinforce effective strategies. Unlike more nuanced aspects of clinical communication, information gathering often follows clear patterns and protocols, making it especially receptive to structured feedback.

The “creating context” subdomain also showed significant improvement, likely influenced by ChatGPT’s specific feedback patterns. The LLM consistently encouraged participants to think deeper into symptom causation, a key component of this subdomain. Specifically, the feedback addressed common oversights in gathering contextual information, such as pre-existing conditions and relevant history like recent travel, helping students develop more comprehensive interviewing strategies.

The absence of significant improvement in the “focusing questions” subdomain can be attributed to a ceiling effect: many participants entered the study with already well-developed skills in this area, as evidenced by five participants achieving maximum scores on the five-point Likert scale for at least one of the first three items. This high baseline performance left limited room for measurable improvement in question-focusing techniques.

### LLM-simulations are a powerful “Add-On” but do not yet replace real human interactions

The self-report data provided by the students underscores a high degree of perceived authenticity and accuracy with their interactions with LLM. This is an important observation, as it relates to the concept of suspension of disbelief in the context of human-LLM interaction [27, 28]. It suggests a willingness to engage with the LLM as if it were a bona fide conversational partner. Moreover, studies have indicated that AI-driven text interactions can closely resemble real human conversations as the language generated by LLMs exhibits many human-like characteristics [29]. This allows students to set aside the awareness that they are communicating with an artificial intelligence. This condition may have enhanced the immersion of the simulation, potentially leading to more engaged learning experiences, and satisfaction.

However, while this suspension of disbelief can lead to satisfying and engaging interactions, it is also important to recognize the limitations of LLM-based conversations. The immersive nature of these interactions may not fully replicate the complexity of real-life conversations. For example, LLM-simulation neglects non-verbal and social aspects [30]. In patient encounters, humans rely heavily on facial expressions, body language, tone of voice, and other subtle cues to interpret meaning and derive clinical decisions [31]. These non-verbal elements often carry as much, if not more, information than the verbal communication themselves. For instance, research has shown that a physician’s ability to read and respond to a patient’s non-verbal cues can significantly impact the quality of care and patient outcomes [30, 31].

Nonetheless, this new tool offers students a potentially unlimited opportunity to prepare for medical conversations in terms of number, time, and variety of case scenarios. Additionally, it serves as a cost-effective alternative to compensate for the high costs associated with simulated patients, as well as the resources needed for real patient interactions and dedicated facilities. In the context of telemedicine and the growing prevalence of patient communication via chat, this tool also provides relevant training opportunities. We want to emphasize that this is intended as a supplementary training resource for medical students and not as a replacement for real-life interactions.

### Limitations of the study

This study has several limitations. First, as previously noted, the study used the “Playground” interface of ChatGPT for its execution. One limitation is that ChatGPT 3.5’s training data only extends up to January 2022. A more significant limitation is the ongoing development of the language model, which may affect reproducibility. For instance, in March 2023, OpenAI updated its model, potentially influencing the consistency of results in future studies. It has not been tested whether the results can be replicated using newer versions.

Second, the conversations with ChatGPT were conducted in German. It can be assumed that the answers, questions and feedback given by ChatGPT are even more precise in English due to the fact that ChatGPT was originally trained on English texts.

Third, although the sample used for this randomized control study is relatively small, a significant effect was discovered. However, future studies will benefit from increasing the statistical power and increase the number of participants. Additionally, future studies should look into how the effect varies across different audiences from different geographic regions, cultures, and contexts.

Forth, we observed that in order to overcome the latency produced by ChatGPT, some participants “learned” to combine several questions into one prompt to get a more comprehensive answer. However, asking multiple questions in a row does not reflect a realistic scenario during medical history conversations. Thus, the use of ChatGPT may not reflect how participants would actually engage in those conversations in real life.

## Conclusion

Our study demonstrated a significant learning effect in medical students through LLM-generated feedback for practicing CDM. These findings contribute to the ongoing, lively discussion about the potential of LLMs in CDM education and medical training overall [32–34], paving the way for individualized learning and cost efficiency. Additionally, we outlined the opportunities and challenges associated with using LLM for patient simulations.

## Electronic supplementary material

Below is the link to the electronic supplementary material.

Supplementary Material 1: Further examples of AI-generated feedback are shown. Feedback quality ranged from specific (as exemplarily shown in Panel A) to unspecific (Panel B), with the majority being specific

## Data Availability

The data that support the findings of this study are available on request from the corresponding author, DD.

## Abbreviations

AI: Artificial Intelligence
ANOVA: Analysis of Variance
AR: Artificial Reality
CDM: Clinical Decision Making
CRI-HTI: Clinical Reasoning Indicator-History Taking Inventory
GPT: Generative Pre-trained Transformer
LLM: Large Language Model
VR: Virtual Reality
