# Supplementary figures and images for: Large language models improve clinical decision making of medical students through patient simulation and structured feedback: a randomized controlled trial
Source: BMC Med Educ. 2024 Nov 28;24:1391. doi: 10.1186/s12909-024-06399-7 (PMC11605890; doi:10.1186/s12909-024-06399-7)

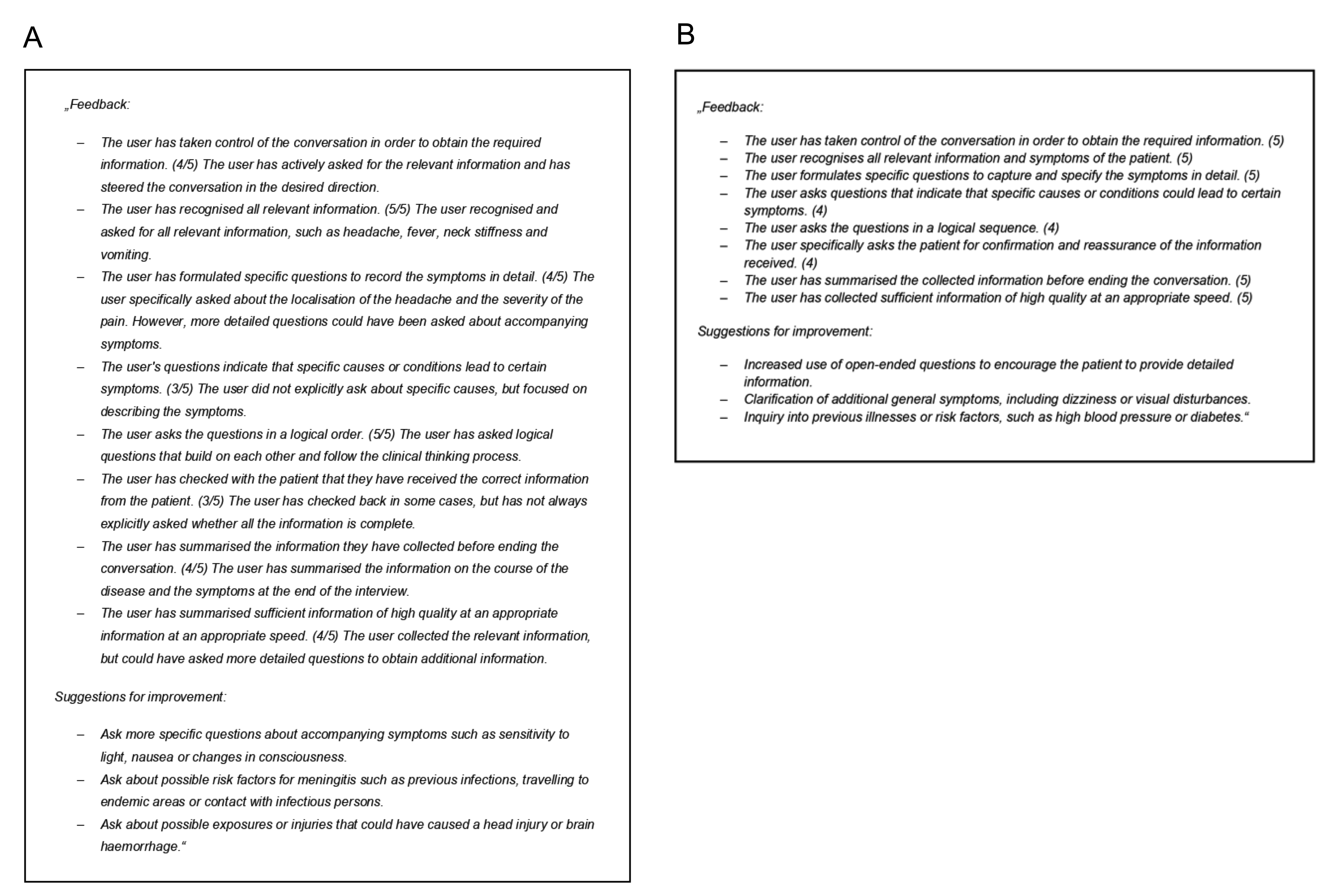

Supplement: Supplementary file 1 — Supplementary Material 1: Further examples of AI-generated feedback are shown. Feedback quality ranged from specific (as exemplarily shown in Panel A) to unspecific (Panel B), with the majority being specific [file 12909_2024_6399_MOESM1_ESM.png]
